# Knowledge, attitudes, and practices of pediatricians in relation to breastfeeding support: A national survey in Lebanon

**DOI:** 10.1371/journal.pone.0281865

**Published:** 2023-04-06

**Authors:** Hiba Al Rifai, Hiba Shatila, Lara Nasreddine, Nadine Yazbeck, Tamar Kabakian-Khasholian, Leila Itani, Farah Naja

**Affiliations:** 1 Faculty of Agricultural and Food Sciences, Department of Nutrition and Food Science, American University of Beirut, Beirut, Lebanon; 2 Human Nutrition Department, College of Health Sciences, QU Health, Qatar University, Doha, Qatar; 3 Department of Pediatrics and Adolescent Medicine, American University of Beirut Medical Center, Beirut, Lebanon; 4 Faculty of Health Sciences, Health Promotion and Community Health Department, American University of Beirut, Beirut, Lebanon; 5 Faculty of Health Sciences, Department of Nutrition and Dietetics, Beirut Arab University, Beirut, Lebanon; 6 Department of Clinical Nutrition and Dietetics, College of Health Sciences, Research Institute of Medical & Health Sciences (RIMHS), University of Sharjah, Sharjah, United Arab Emirates; 7 Faculty of Agricultural and Food Sciences, American University of Beirut, Beirut, Lebanon; Faculty of Medicine, Saint-Joseph University, LEBANON

## Abstract

**Background:**

Rates of breastfeeding (BF) remain suboptimal despite overwhelming evidence for its benefits to the mother and child. Pediatricians play an important role in supporting breastfeeding (BF). In Lebanon, the rates of both exclusive and continued BF are critically low. The objective of this study is to examine the knowledge, attitudes and practices (KAP) of Lebanese pediatricians in relation to supporting BF.

**Methods:**

A national survey of Lebanese pediatricians was conducted through Lime Survey (n = 100, response rate 9.5%). The list of pediatricians’ emails was obtained from the Lebanese Order of Physicians (LOP). Participants completed a questionnaire covering, in addition to sociodemographic characteristics, the KAP, related to supporting BF. Descriptive statistics and logistic regressions were used in data analysis.

**Results:**

The most prevalent gaps in knowledge were related to the positioning of the baby during BF (71.9%) and the association between the mother’s fluid intake and her milk production (67.4%). With regards to attitudes, 34% of participants reported unfavorable attitudes towards BF in public and BF while working (25%). As for practices, more than 40% of pediatricians kept formula samples and 21% had formula-related advertising in their clinics. Half of the pediatricians reported rarely/never referring mothers to lactation consultants. After adjustment, both being a female pediatrician and having done the residency in Lebanon were significant predictors of better knowledge (OR = 4.51 (95%CI: 1.72–11.85) and OR = 3.93 (95%CI: 1.38,11.19) respectively.

**Conclusion:**

This study revealed important gaps in the KAP related to BF support among Lebanese pediatricians. Coordinated efforts ought to be exerted to educate and equip pediatricians with needed knowledge and skills to support BF.

## Background

The first 1000 days of life have been increasingly postulated as the foundation of the child’s lifelong growth, health and wellbeing [1]. Breastfeeding (BF) is recommended exclusively for the first six months of life [2]. After six months, nutritious complementary foods should be introduced while BF is continued for two years and beyond [2]. Despite the well-established benefits of BF, worldwide rates are still suboptimal [3]. Globally, only 43% of newborns receive BF during the first hour of birth, and only 41% of infants (0–6 months) are breastfed exclusively. Furthermore, even though most mothers (70%) provide breastmilk during the first year of life, only 45% continue to do so by the age of two years [3]. These estimates are far below the global goals for BF as set by the WHO and UNICEF in the 2019 Global Score Card, whereby the rates of BF should increase to reach at least 70% initiation within the first hour, 70% exclusive BF in the first 6-months, 80% of BF at one year, and 60% at two years of age [3].

To achieve such improvement in BF indicators, it is essential to examine the reasons behind the existing low rates of BF. According to the conceptual framework proposed by the latest Lancet series for the determinants of BF decision, three main key set of factors are implicated: the individual, the setting, and the structure [4]. An integral component of ‘structural’ factors is the role played by healthcare providers [4]. Of health care providers, pediatricians are considered among the most influential regarding the feeding method of babies [5]. Pediatricians–being frequently visited throughout the first year of the baby’s life–have more than one opportunity to advise regarding the feeding method and support BF [6]. Their role includes providing evidence-based information, investigating possible barriers and enablers to BF, managing common BF related problems, identify when to recommend supplementation or not, suggesting ways to prevent unnecessary supplementation and weaning and eventually working with the mother to lay out a patient-centered plan in order to maximize the possibility of adherence to BF recommendations [7]. Several studies showed that mothers choose to breastfeed, continue to BF, or even switch from formula to BF because of the positive influence of their pediatricians [5].

Lebanon is a middle-income Middle-Eastern country with suboptimal rates of BF [8]. More specifically, exclusive BF of 1-month-old infants is 40%, and of 4–5 months old infants is only 2%. Moreover, mixed feeding during the first month of age is prevalent (more than 40%). As for continued BF, 41.8% of babies continue BF—with the complimentary food—up to 9 months, 37.5% up to 15 months, and only 14.6% up to 23 months [8–11].

In light of the critical role of pediatricians in supporting BF and the dire need to improve the current BF rates in Lebanon, the main aim of this study is to examine the knowledge, attitudes, and practices (KAP) of Lebanese pediatricians in relation to supporting BF. A secondary objective is to explore the sociodemographic correlates of the knowledge of Lebanese pediatricians.

## Methods

This is a cross-sectional survey, conducted between December 2019 and August 2020 in Lebanon. The practicing Lebanese Pediatricians constituted the target population. Data collection was conducted using an online survey sent via email. The list of emails was obtained from the Lebanese Order of Physicians (LOP). All physicians in Lebanon have to be registered at LOP before they can practice their profession. Email invitations were followed with periodic reminders for pediatricians to complete the questionnaire. The study protocol and tools were reviewed and approved by the Institutional Review Board (IRB) at the American University of Beirut. The voluntary nature of the study was highlighted in the invitation. Pediatricians were asked to read a consent form and agree to it before completing the questionnaire. The questionnaires were received anonymously through the lime survey system where a random identification number was generated for each participant. Data files were securely saved in a password-protected laptop. Sample size calculations showed that, in order to estimate a prevalence of 50%, with 95% confidence and ±10% margin of error a total of 90 participants is needed [12]. Of the 1383 registered pediatricians, emails were available for 1056 physicians. Of those, 131 completed the questionnaire. Out of these responses, 31 questionnaires were missing answers on all of the three main sections (knowledge, attitudes, practices) and were not included in the analysis, hence the number of responses included in the study was 100. As such the response rate in the study was 100/1056 (9.5%).

The questionnaire used in this study was adapted from that used by Pound et al. for the examination of BF knowledge, confidence, beliefs, and attitudes among Canadian physicians [13]. In developing their questionnaire, Pound et al. [13] used a multitude of sources including: 1) thorough research of existing literature, 2) inputs from International Board Certified Lactation Consultants, 3) the American Academy of Pediatrics (AAP) Position Statement on “Breastfeeding and the Use of Human Milk, and 4) the Registered Nurses’ Association of Ontario Self-Learning Module on Breastfeeding. For the purpose of this study, the questionnaire developed by Pound et al. was adapted to the local context by a panel of experts consisting of 1) a clinical pediatrician, 2) a nutrition epidemiologist, 3) a human nutritionist, 4) a public health specialist, and 5) a BF and lactation specialist. The panel examined the content validity and clarity of the questionnaire as well as its adequacy addressing the research objectives. The questionnaire was developed in English, then it was translated to Arabic. To ensure parallel form reliability, the questionnaire was back translated from Arabic to English. The English translated and the original version of the questionnaire were then compared for consistency. Both the Arabic and the English forms of the questionnaire were pilot tested on a convenient sample of 10 pediatricians. As a result of this pilot testing a few questions were amended. For example, the questions related to the pediatricians’ private experience (if they breastfed their babies) and those related to HIV were deleted, as these were perceived culturally inappropriate. Data collected from the pilot testing phase were not included in this study. The final version of the questionnaire (Appendix I) consisted of four main sections (Demographics, Knowledge, Attitudes, and Practices). The first section addressed the demographic and work-related characteristics of the participants. The knowledge section explored the pediatrician’s knowledge, using 15 True and False questions (T/F) and 7 Multiple Choice Questions (MCQs). The questions included in this section refer to mothers with normal conditions rather than clinical cases. The T/F questions addressed several topics: BF duration, solid foods introduction, water supplementation during the night, the nutrition value of infant formulas and how they compare to breastmilk, the use of bottles for pumped milk, the effect of supplementing with formula on the success of BF, the effect of exercise on breastmilk, the effect of fluids on milk supply, the importance of BF on Sudden Infant Death Syndrome (SIDS) and ovarian and breast cancer prevention in babies and mothers respectively, the contraindications of BF, and the effectiveness of breastmilk after the age of two. As for the MCQs, they covered topics such as the proper tongue position during BF, the signs of correct latching, the management of sore nipples, the best way to address frequent feedings in young infants, the proper advice for a jaundiced newborn, exclusive BF, and the adequate timing for BF initiation. A total knowledge score was computed by adding the points on the 22 questions included in this section of the questionnaire. Correct answers were given 1 point, whereas wrong answers as well as “I don’t know” answers were given 0 points. As such the knowledge scores ranged between 0 (minimum score) and 22 (maximum score); higher scores reflected better knowledge.

The section on attitudes/ beliefs of pediatricians in relation to BF included 15 questions. Pediatricians were asked to record their level of agreement, using a 5-point Likert scale ranging from strongly agree to strongly disagree, on issues related to BF and formula feeding such as the role of pediatricians in assessing BF, their influence on the mothers’ decision to breastfeed, and their perception on mixed feeding and formula feeding (9 questions). The attitudes section also included 6 questions related to the confidence/comfort of pediatricians in teaching and counseling mothers on BF issues and assessing and addressing BF problems. The pediatricians indicated their confidence/comfort on these topics using a 5-point Likert scale ranging from very confident/ comfortable to very unconfident/ uncomfortable. The section on practices examined the pediatricians’ BF counseling and support (12 questions). The first 5 questions addressed the frequency of: counseling mothers on BF, encouraging BF after mothers return to work, referring mothers to lactation consultants, and recommending formula feeding. These questions were rated on a 5-point Likert scale from (5): always or almost always to (1): never or almost never. For the remaining 7 questions, pediatricians were asked to indicate (Yes) or (No) on several statements concerning their practices regarding formula advertising, formula supplementation, BF promotion and recommendation.

### Statistical analysis

The Statistical Package for Social Sciences (SPSS, version 23) was used in data analysis. Descriptive Statistics (frequencies and percentages) were used to summarize the pediatricians’ characteristics, as well as their practices, attitudes and knowledge. In order to examine the correlates of the knowledge, simple and multiple logistic regression analyses were carried out. The dependent variable in these analyses was a dichotomous variable (good versus poor knowledge) with the mean knowledge score used as cutoff. The sociodemographic and work-related characteristics constituted the independent variables. Factors that showed significance at a p-value < 0.05 in the simple regression were entered into the multiple regression. Statistical significance was established at a p-value < 0.05.

## Results

Table 1 represents the sociodemographic characteristics of the study participants. The majority of pediatricians were above 41 years old (74.5%) and had more than 15 years of experience (66.7%). The study sample consisted of slightly more females (58.8%) than males (41.2%). (Table 1).

**Table 1:** Characteristics of study sample.

Pediatrician’s Characteristics	n	%
**Age**		
< 41 years	25	25.5
41–50 years	28	28.6
> 50 years old	45	45.9
**Gender**		
Male	40	41.2
Female	57	58.8
**Residency place**		
In Lebanon	67	69.8
Abroad (Outside Lebanon) *	29	30.2
**Work Area**		
North (Akkar and North)	9	9.1
Bekaa (Baalbeck-Hermel and Bekaa)	5	5.1
Beirut	33	33.3
Mount Lebanon	42	42.4
South (Nabatieh and South)	10	10.1
**Workplace**		
Clinic	67	67.7
Hospital	29	29.3
Others **	3	3.0
**Duration of Practice (years)**		
0 to 5	16	16.2
6 to15	17	17.2
>15	66	66.7
**Learned about BF through own experience**		
No	55	55.6
Yes	44	44.4
**Learned about BF through Self-directed learning**		
No	49	49.5
Yes	50	50.5
**Learned about BF through Medical school**		
No	45	45.5
Yes	54	54.5
**Learned about BF through Residency**		
No	40	40.4
Yes	59	59.6
**Had certification in BF support** ***		
No	90	91.8
Yes	8	8.2

Total numbers in this table are not always equal to 100 (number of participants in this study) because of missing answers.

* France, Belarus, Syria, Jordan, Lithuania, Poland, Russia, US, UK.

** NSSF, hospital and clinic.

***Any certification in breastfeeding support including, but not limited to, the International Board Certified Lactation Consultant (IBCLC), the Fellow of the Academy of Breastfeeding Medicine (FABM), the Certified Lactation counselor (CLC) certification.

The results of the self-evaluation of the participants’ knowledge are presented in Table 2A (true/false/I don’t know questions) and 2B (MCQs). Most pediatricians answered correctly the questions related to the recommendations of BF initiation (91%), exclusivity (93.3%) and duration (83.1%). However, around 67.4% of the participants believed that increasing mother’s fluid or milk intake will increase her milk production and 36% of pediatricians did not know the recommendations regarding the time of solid foods introduction. (Table 2A). Many indicators of a proper latch were missed by the surveyed pediatricians. (Table 2B).

**Table 2:** a. Evaluation of self-knowledge towards BF among study participants: True False Questions. **b.** Evaluation of self-knowledge towards BF among study pediatricians: Multiple Choice Questions*.

	Correct Answer	Answered correctly		Answered incorrectly or I don’t know	
		n	%	n	%
Exclusive BF is recommended for the first six months of life by the World Health Organization (WHO) **[14]**	True	83	93.3	6	6.7
It is recommended to completely wean breastfed babies from the breast at the age of 1 year **[15]**	False	74	83.1	15	16.9
Solid foods introduction should start when the baby is 4 months according to WHO **[15]**	False	57	64	32	35.9
Offering the baby water during the night will help the baby hydrate and sleep better **[16]**	False	84	94.4	5	5.6
The current infant formulas are nutritionally equivalent to breastmilk **[17]**	False	68	76.4	21	23.6
Formula is sometimes superior to breastmilk if it was fortified with iron and vitamin D that are both lacking in human milk **[17]**	False	72	81.8	16	18.2
Bottles are the best option to use when breastmilk is pumped or when formula is provided **[14]**	False	34	38.6	54	61.4
Supplementing with formula in the first weeks of life will not affect BF success **[14]**	False	81	91	8	9
Moderate exercise in the mother can decrease quality and quantity of breastmilk **[18]**	False	79	88.8	10	11.2
Increasing mother’s fluid or milk intake will increase her milk production **[19]**	False	29	32.6	60	67.4
BF has been shown to decrease the risk of SIDS **[20]**	True	78	87.6	11	12.4
BF is contraindicated in mothers with Hepatitis C **[14]**	False	51	57.3	38	42.7
BF decreases the risk of ovarian and breast cancer in mothers **[14]**	True	85	95.5	4	4.4
BF is safe to continue in mothers who have herpes simplex on a breast as long as the child only feeds from the unaffected breast **[21]**	True	68	76.4	21	23.6
Breastmilk loses its core components after the baby is two years old **[22]**	False	44	49.4	45	50.6

	Answered correctly		Answered incorrectly or I don’t know	
	n	%	n	%
Exclusive BF consists of: **Only breast milk, no other liquids or solids, not even water, with the exception of vitamins, minerals supplement, medicines or Oral Rehydration Solutions. [14]**	73	82	16	18
For a baby who is BF effectively, the tongue is: **Down and over the gum line [14]**	60	67.4	29	32.6
When a BF mother complains that her nipples are sore, the first thing to do is: **Assess baby’s position and latch [14]**	54	60.7	35	39.3
All of the following are signs that a baby is latched on properly except: **No part of the areola can be seen [14]**	25	28.1	64	71.9
A mother complains that her six-week-old infant has been BF almost every hour for a day or two: **Explain that the baby requires more milk because he/she is growing and frequent BF is his/her way to increase milk supply [14]**	47	52.8	42	47.2
An otherwise healthy 5-day old breastfed infant is admitted to the hospital with jaundice. In addition to treating the child with phototherapy, the first thing to do is: **Recommend more frequent BF sessions, and teach mother how and when to use a breast pump [14]**	74	83.1	15	16.9
BF initiation should be: **Directly after birth [14]**	81	91.0	8	9

Total numbers in this table are not always equal to 100 (number of participants in this study) because of missing answers.

* The text in bold indicates the correct choice for each of the questions.

1World Health Organization (WHO). Geneva, Switzerland: WHO; 2009 [cited 2021 Oct 6]. Infant and young child feeding: Model chapter for textbooks for medical students and allied health professionals. Available from: https://apps.who.int/iris/handle/10665/44117.

Table 3A and 3B summarize the attitudes of the study participants towards BF. The majority of pediatricians had positive attitudes towards their responsibility to evaluate and follow up on BF (Table 3A). On the other hand, a few negative attitudes towards BF were identified, especially regarding the practicality of breastfeeding in public and stopping BF once the mother knows she is pregnant. (Table 3A).

**Table 3:** a. Attitudes towards BF among study participants. **b.** Self-assessment of pediatricians’ confidence/ comfort in addressing and managing BF-related problems.

	Strongly Agree		Agree		Neutral		Disagree		Strongly Disagree	
	n	%	n	%	n	%	n	%	n	%
The child’s pediatrician is responsible for performing an evaluation of BF, including position, latch and milk transfer in the first 3 to 5 days after birth	50	50	17	17	17	17	12	12	4	4
As a pediatrician, I have an influence on a mother’s decision to breastfeed her infant	66	66	20	20	10	10	3	3	1	1
It is practical for working mothers to continue to breastfeed their infants	46	46	29	29	20	20	2	2	3	3
It is acceptable for women to breastfeed in public	45	45	21	21	21	21	6	6	7	7
My residency training prepared me to support BF mothers	52	52	13	13	16	16	11	11	8	8
It’s not the pediatrician’s responsibility to follow up on BF progress and success	3	3	3	3	9	9	15	15	70	70
Mixed feeding (breastmilk and formula) is a more practical acceptable feeding method	4	4	7	7	19	19	19	19	51	51
Formula-fed babies are just as healthy as breastfed babies	5	5	7	7	21	21	22	22	45	45
A mother should stop BF once she knows she is pregnant	21	21	7	7	14	14	16	16	42	42

	Extremely confident		Very Confident		Moderately Confident		Slightly Confident		Not at all	
	n	%	n	%	n	%	n	%	n	%
Teaching mothers about good BF positioning	68	68.7	21	21.2	5	5.1	2	2	3	3
Addressing BF related problems (engorgement, mastitis, etc.)	67	67	21	21	7	7	3	3	2	2
Evaluating whether a baby’s latch is successful	56	56.6	20	20.2	16	16.2	2	2	5	5.1
Assessing whether there is good milk transfer from mother to baby during BF	58	58	16	16	15	15	7	7	4	4
Counselling mothers on cracked and sore nipples	62	62	26	26	7	7	3	3	2	2
Teaching mothers how to express milk	60	60	19	19	13	13	5	5	3	3

Total numbers in this table are not always equal to 100 (number of participants in this study) because of missing answers.

Table 4A shows the pediatricians’ self-reported frequency of BF-related practices. The majority of pediatricians (86.9%) reported asking BF mothers how BF is going throughout the first year of their infants’ life. Almost 89% reported always recommending women to continue BF after returning to work. Meanwhile, only 27% of pediatricians said that they sometimes ask BF mothers to breastfeed their infants in front of them so that they can assess the feeding, and 37.4% rarely or never do so. (Table 4A). Table 4B summarizes the second set of the pediatricians’ practices results, to which pediatricians answered with either yes or no. While almost 40% of pediatricians kept formula samples in their clinics to distribute to mothers and their babies, only 25.3% had brochures/ pamphlets in their clinics to give to mothers about BF resources in the city. Additionally, more than 20% of pediatricians reported to have formula-related advertising in their clinics. (Table 4B).

**Table 4:** a. Practices related to supporting BF among pediatricians participating in the study (frequency using a 5-point Likert scale). **b.** Practices related to supporting BF among pediatricians participating in the study (frequency using Yes or No). (n = 99).

	Always		Often		Sometime		Rarely		Never	
	n	%	n	%	n	%	n	%	n	%
Ask BF mothers how BF is going in the first year of their infants’ life	86	86.9	9	9.1	2	2	0	0	2	2
Ask BF mothers to breastfeed their infants in front of you so that you can assess the feeding	17	17.2	18	18.2	27	27.3	19	19.2	18	18.2
Recommend women to continue BF after returning to work	88	88.9	7	7.1	2	2	0	0	2	2
Refer mothers to lactation consultants to address BF problems	12	12.1	18	18.2	20	20.2	22	22.2	27	27.3
Recommend formula feeding (excluding special needs)	0	0	6	6.1	8	8.1	21	21.2	64	64.6

	Yes		No	
	n	%	n	%
Do you keep samples of formula in your clinic to distribute to mothers and their babies	39	39.4	60	60.6
Do you have advertisement in your clinic on formula products, or well-baby care documents that have formula companies’ logo on them?	21	21.2	78	78.8
Do you have brochures / pamphlets in your clinic that you give to mothers about BF resources in the city	25	25.3	74	74.7
Do you recommend to start supplementing with formula, if a healthy term baby who is exclusively breastfed has not regained birth weight by 2 weeks	31	31.3	68	68.7
Do you routinely recommend to supplement with formula if a mother feels that her milk supply is inadequate	26	26.3	73	73.7
Do you typically recommend feeding glucose water or formula to the otherwise healthy newborn while waiting for the mother’s milk to come in	15	15.2	84	84.8
Do you routinely recommend that babies breastfeed on each side for 15–20 minutes every 3 hours	74	74.7	25	25.3

Total numbers in this table are not always equal to 100 (number of participants in this study) because of missing answers.

The mean (± SD) of the knowledge score of all pediatricians was 15.74/22 (± 3.2). The minimum score was 6 while the maximum was 22. After adjustment using multiple linear regression, both being a female and having done the residency in Lebanon were significant correlates of better knowledge (OR = 4.51 (95%CI: 1.72, 11.85) and OR = 3.93 (95%CI: 1.38, 11.19) respectively). (Table 5)

**Table 5:** Predictors of knowledge score related to BF support among study participants, as examined by simple and multiple logistic regression.

	Knowledge score (mean SD)	OR (95% CI)	Adjusted OR (95% CI)
**Pediatricians Age (years)**			
Less than 41 years old	16.26 ±2.93	Ref	-
41 to 50 years old	16.08 ±2.12	1.15(0.37, 3.64)	-
More than 50 years old	15.18± 3.91	0.73(0.26,2.06)	-
**Gender**			
Male	14.79 ±3.03	Ref	-
Female	16.67± 3.08	**4.16(1.68,10.31)**	**4.51(1.72,11.85)**
**Residency place**			
Abroad (outside Lebanon)	14.54 ±2.90	Ref	-
Lebanon	16.30 ±3.16	**3.26(1.25,8.55)**	**3.93(1.38, 11.19)**
**Major Working Area**			
North (Akkar and North)	13.67 ±3.00	Ref	-
Bekaa (Baalbeck-Hermel and Bekaa)	17.25 ±2.75	10.5(0.67,165.11)	-
Beirut	15.52 ±3.47	4.96(0.87, 28.15)	-
Mount Lebanon	16.47 ±3.14	4.90(0.89, 26.97)	-
South (Nabatieh and South)	15.00 ±2.36	3.50(0.47,25.90)	-
**Major workplace**			
Clinic	15.95 ±3.15	Ref	-
Hospital	15.46 ±3.42	0.92(0.36, 2.32)	-
Other	14.00 ±3.00	0.39(0.03, 4.59)	-
**Years of Practice**			
0 to 5	16.00 ±3.28	Ref	-
6 to15	16.40 ±2.26	1.13(0.26,4.894	-
>15	15.51 ±3.42	0.83(0.26, 2.69)	-
**BF Background**			
Own experience and Self-directed learning			
No	15.53 ±2.76	Ref	-
Yes	15.84 ±3.45	1.08(0.45, 2.61)	-
Medical school and Residency			
No	15.79 ±3.81	Ref	-
Yes	15.72 ±3.07	0.84(0.30, 2.35)	-
**Certification in BF support**			
No	15.66 ±3.06	Ref	-
Yes	16.63 ±4.84	1.40(0.32,6.24)	-

*The adjusted model included the variables that were significantly associated with knowledge in the simple regression analyses, and which were ‘gender’ and ‘place of residency’.

## Discussion

To the best of our knowledge, this is the first study, in Lebanon and in the Middle East and North African region, to investigate the KAP of practicing pediatricians in relation to supporting BF. The results of this study revealed several gaps and opportunities regarding the BF KAP of Lebanese pediatricians and identified the sociodemographic predictors of the pediatricians’ knowledge.

A noticeable proportion of participants in this study were knowledgeable about several BF topics. In agreement with the available literature, the participants in this study were able to identify the protective effects of BF on SIDS among babies and on ovarian and breast cancer among mothers [13,23]. Most pediatricians in this study were aware of the recommendations of BF initiation, exclusivity and duration. These findings can be attributed to the recent BF campaigns in Lebanon which might have made Lebanese pediatricians more interested in seeking updated BF information.

The study uncovered disconcerting misconceptions among the pediatricians mainly in what relates to BF contraindications and formula milk. Such myths/misinformation can lead to recommending early cessation of exclusive BF or to unnecessary supplementation of formula. Despite the well-established evidence against the use of bottles to provide pumped or supplemented milk [24], most pediatricians in this study lacked this knowledge. In addition, the majority of pediatricians in this study either thought that breastmilk loses its core components after the baby is two years old or did not have knowledge on this issue. This indicates that pediatricians are not aware of the updated evidence regarding the continuation of BF” as long as the mother and the baby want beyond two years of age” [2], and the long term advantage of breastmilk in reducing obesity risk and enhancing immunity [25]. Suboptimal knowledge related to the assessment and management of BF problems was also identified among the study participants. Similar knowledge gaps have been reported in a previous study among medical students in the Lebanese Public School of Medicine [23]. These deficiencies could stem from the limitations of the medical curriculum to address BF related topics and the lack of directed continuous education programs. This highlights the role of professional associations in actively pursuing updating the pediatricians on the recent BF recommendations. Accordingly, interventions to address these knowledge gaps are warranted throughout the pediatricians’ education and practice.

A general positive attitude towards BF was shown among pediatricians in this study. The majority of the study participants believed that they are responsible for the evaluation and follow up of early and continued BF. Additionally, they acknowledged their influence on the mother’s decision to breastfeed. This comes in line with studies exploring maternal views of BF in Lebanon where physicians were considered among the highest influencers on BF decisions [10,26]. Moukarzel et al. [23] have also confirmed a general positive attitude towards BF among residents in the Lebanese Public School of Medicine. Furthermore, the study participants reported confidence/ comfort in BF assessment, counselling, and management. However, when comparing these attitudes to the relating knowledge, the pediatricians in this study were not able to identify the signs of proper BF and the management of sore nipples. The reported confidence among study participants could be due to ‘not knowing what one does not know’. In any case, the limited knowledge observed in this study constitutes an opportunity for improvement given the pediatricians’ positive attitudes. Moreover, it highlights a risk of mismanagement and mis-advising on BF problems and thus threatens BF success. Nonetheless, the sense of accountability among pediatricians is a promising starting point and an opportunity to foster collaboration with the pediatricians in order to address their gaps in KAP related to BF support.

Unfavorable attitudes towards BF among the study participants were identified at several levels. The study participants had negative attitudes towards BF in public, and towards the practicality of BF for working mothers. BF in public can be fairly related to the conservative culture in Lebanon where BF is still considered a private act.

Consistent with the available literature, the findings of this study highlighted an acceptance of mixed feeding among surveyed pediatricians where they have agreed to its practicality [27,28]. In addition, one third of the participants had either neutral attitudes or agreed that formula fed babies are as healthy as breastfed babies. In line with this finding, a study by Quinn and Tanis [29] showed that pediatricians were less likely to believe that infants who breastfeed are healthier than formula-fed infants. Such findings suggested a positive attitude towards formula feeding among participants. The latter could be a result of the efforts exerted by the Breastmilk Substitute (BMS) industry efforts to influence pediatricians’ attitudes starting early during their residency training where they get exposed to targeted marketing of formula products [30].

Even though BF during pregnancy has been documented as safe with no effects on delivery or birthweight [31], many pediatricians in this study believed that mothers should stop BF once they know they are pregnant. This belief can make pediatricians recommend against BF once a mother becomes pregnant and hence babies lose their right of optimal nutrition. This is worrying especially among mothers in the region who choose to give birth with short inter pregnancy intervals [32].

In agreement with previous reports, the findings of this study revealed that some pediatricians did not think their residency training has prepared them well to support BF [13,27]. As discussed by Freed et al. [33], this can be attributed to the didactic nature of training where residents are not exposed to an actual clinical setting to develop the required BF support skills. This was further confirmed by Feldman-Winter et al. [34] and Hillenbrand and Larsen [35] who have documented that incorporating interactive sessions in the residency curricula can lead to improvements in the pediatricians’ knowledge, confidence, and practices as well as BF rates among mothers visiting the intervention groups.

The study pediatricians reported several good practices related to BF. The vast majority of pediatricians: 1) discussed BF with mothers during the first year, 2) recommended BF for working mothers, and 3) did not recommend feeding glucose water or formula to the otherwise healthy newborn while waiting for the mother’s milk to come in. Despite these good practices, several areas of improvement among the pediatricians were apparent. Concordant with the findings by Pound et al. [13], pediatricians in this study did not routinely ask BF mothers to breastfeed their babies in front of them to assess the feeding. This can be explained by the mother-pediatrician relationship where the mother is not the pediatrician’s patient (the baby is) and hence asking her to breastfeed might not be culturally conventional especially if the pediatrician is a male. Despite this, more than half of the participants rarely or never referred mothers to lactation consultants to address BF problems. Such a low referral rate could be due to the small number of certified lactation consultants in the country. For instance, according to the International Board of Lactation Consultant Examiners, there are only 10 IBCLCs in Lebanon. [36] These results suggest that mothers might be missing the opportunity to be observed–by either the pediatrician or the lactation consultant–while BF in order to assess and evaluate the feeding.

Furthermore, a significant number of pediatricians in this study kept formula samples, had formula advertising in their clinics, or used documents with formula logo on them. This is consistent with previous records highlighting the distribution of free formula samples by Lebanese pediatricians [37,38] and the breaches of the Law 47/2008 that happened before and after its ratification [39]. Promotion by the BMS industry, social pressure and the mother’s demand to formula feed her baby were reported as reasons for promoting formula by pediatricians [30]. These practices are worrying as mothers become exposed to a formula-friendly environment instead of a BF-friendly one endangering the exclusivity and continuation of BF [40].

These formula conducive practices can be exacerbated by the overwhelming majority of pediatricians who reported to not have brochures or pamphlets to give to mothers about BF resources in the city. This indicates that the chances of a mother leaving the clinic with a formula sample are much higher than those of a mother leaving the clinic with BF support materials. Pediatricians may be unaware of the available BF resources, however, it is important to equip all pediatric clinics with these information to disseminate to mothers. In Lebanon there are multiple support groups (online and face to face), Non-Governmental Organizations (NGOs), many BF consultants, in addition to the hotline of the Ministry of Public Health (MOPH) all of which can support mothers and address their BF concerns. These resources if utilized by mothers were shown to be effective in increasing the exclusivity and continuation of BF [41]. This issue was observed among American and Canadian pediatricians where the majority did not refer mothers to community support groups and did not provide them with written resources, respectively [13,42].

Even though, only 15% of the study participants indicated that they would recommend formula feeding (excluding special needs) often or sometimes, a noticeable number of pediatricians reported to recommend formula supplementation if a mother feels her milk is inadequate or if the healthy baby does not regain birthweight by 2 weeks. These two conditions are not considered “special needs” and hence supplementing with formula is unnecessary and might impede successful BF [43]. Such recommendations can jeopardize the success of BF even if the mother wants to breastfeed [6].

The majority of Lebanese pediatricians in this study recommended timed feeding. This comes in alignment with the knowledge assessment results highlighting a significant number of pediatricians who did not know how to address a breastmilk-demanding child. The problem with these recommendations is that they are often unrealistic where a baby demands BF on a much smaller interval [44]. Thus, when a mother is advised to breastfeed every three hours and faces a demanding baby she might feel her milk supply is inadequate or the baby might not gain enough weight. As discussed previously, a significant number of pediatricians would recommend supplementing with formula for these particular reasons. It is, therefore, recommended to update pediatricians on the importance of BF on demand (as much as the child wants day and night) in light of the latest international recommendations [2].

The regression analyses identified few significant predictors of the knowledge scores. Female pediatricians were more likely to have better knowledge. This has been consistently reported in studies worldwide [13,33,45]. Moreover, Al-Sahab et al. [9] found that Lebanese mothers were 1.5 times more likely to continue BF until four months of age if their pediatrician was a female. This can be related to the fact that females are more interested in BF and more comfortable discussing BF issues due to their personal experience [13,33]. Another predictor of better knowledge was having done the residency in Lebanon. Despite the fact that some international programs may carry more information about breastfeeding compared to the Lebanese curriculum, having done the residency in Lebanon could have contributed to a better exposure of the pediatricians to the Lebanese cultural context as well as the expectations of the mothers, therefore better equipping them with the knowledge needed to support BF.

This is the first study to investigate the BF KAP among any group of health professionals in Lebanon, including pediatricians. It constitutes a baseline for the contextualization of the role of healthcare providers in BF support and promotion.

That said, the findings of this study ought to be considered in light of the study limitations and strengths. First, the limited response rate. Despite the maximized efforts to reach the largest possible number of pediatricians, only 9.5% responded. It is well documented that physicians are a hard to recruit group [46] possibly because of their busy schedules [43]. This low response rate can also be attributed to the major instabilities (economic, political, and pandemic-COVID-19) in the country which might have prevented pediatricians from actively participating in the study. That said, the study sample also included participants from across Lebanon in proportions close to those of the national distribution.

This study depended on self-selection and hence those who responded (the 12.4%) might be those most interested in the topic of BF suggesting a possible selection-bias. Accordingly, responders might be more knowledgeable of the topic compared to other pediatricians (non-participants). Therefore, the study findings might constitute a “best case scenario” of the pediatricians’ KAP when compared to the national trends.

## Conclusions

In summary, the dynamic relationship between the knowledge, attitudes, and practices of pediatricians in relation to supporting BF, suggests that efforts are needed to address these KAP components in order to have good knowledge, positive attitudes, and good practices. Hence, it is advised to design multi-component interventions that improve the knowledge related to BF, highlight the importance of acquiring positive attitudes towards BF, and equip the pediatricians with the necessary skills to exhibit BF-friendly practices.

Improving the KAP of pediatricians in relation to supporting BF and expanding the research on this topic, require a multi-sectoral involvement. Health promotion experts, nutritionists, physicians, policy experts, NGO workers, academicians, hospital administrations, LOP, Lebanese Pediatric Society, MOPH and policy makers all need to pool efforts in creating opportunities for pediatricians to support mothers and advocate for a BF-friendly environment where mothers feel safe and supported to initiate and continue BF. Qualitative research is required to complement these findings to capture an in depth understanding of the reasons behind the reported attitudes and practices. Furthermore, studies that investigate the mothers’ perspectives regarding the pediatricians’ practices and recommendations are warranted to better characterize the patient-doctor relationship and dynamics as related to BF.

## Supporting information

S1 Checklist STROBE statement—checklist of items that should be included in reports of *cross-sectional studies*. (PDF)

S1 Appendix Questionnaire used in data collection. (DOCX)

S1 Dataset De-identified dataset used in this study. (RAR)

## Abbreviations

AAP: American Academy of Pediatrics
BF: Breastfeeding
BMS: Breastmilk Substitute
CI: Confidence Interval
KAP: Knowledge, Attitudes, and Practices
LOP: Lebanese Order of Physicians
MCQs: Multiple Choice Questions
MOPH: Ministry of Public Health
NGOs: Non-Governmental Organizations
SIDS: Sudden Infant Death Syndrome
T/F: True and False
WHO: World Health Organization
