# Supplementary material for: Knowledge, attitudes, and practices of pediatricians in relation to breastfeeding support: A national survey in Lebanon
Source: PLoS One. 2023 Apr 6;18(4):e0281865. doi: 10.1371/journal.pone.0281865 (PMC10079043; doi:10.1371/journal.pone.0281865)
Supplement: S1 Appendix — (DOCX) [file pone.0281865.s002.docx]

**Demographics:**

1. **How old are you?**
2. < 41 years old
3. 41 to 50 years old
4. > 50 years old
5. **What is your gender?**
6. Male
7. Female
8. **Did you do your residency training in Lebanon?**
9. Yes
10. No; please specify: ______________
11. **Which governorate do you spend most of your time working in? (please select only one answer)**
12. North (Akkar and North)
13. Bekaa (Baalbeck-Hermel and Bekaa)
14. Beirut
15. Mount Lebanon
16. South (Nabatieh and South)
17. **Is the majority of your work spent in: (please select only one answer)**
18. Private Clinic
19. Public Clinic
20. Private hospital
21. Public hospital
22. Private Teaching hospital
23. Public Teaching hospital
24. Other; please specify: ______________
25. **How many years have you been in practice?**
26. 0 to 5
27. 6 to10
28. 11 to 15
29. > 15 years
30. **Where did you learn about breastfeeding? (please circle all that apply)**
31. Own experience
32. Medical school
33. Residency
34. Self-directed learning
35. Other, please specify: _________________________
36. **Do you hold a certification in breastfeeding support? (e.g. the International Board of Lactation Consultants, online training, hospital based training)**
37. Yes , please specify: _________________________
38. No

**Practices:**

1. **How often do you practice each of the below: please rate from (5) Always or almost always to (1) Never or almost never**

| In the Last 12 months, how often did you: | (5) | (4) | (3) | (2) | (1) |
| --- | --- | --- | --- | --- | --- |
| Ask breastfeeding mothers how breastfeeding is going in the first year of their infants’ life |  |  |  |  |  |
| Ask breastfeeding mothers to breastfeed their infants in front of you so that you can assess the feeding |  |  |  |  |  |
| Recommend women to continue breastfeeding after returning to work |  |  |  |  |  |
| Refer mothers to lactation consultants to address breastfeeding problems |  |  |  |  |  |
| Recommend formula feeding (excluding special needs) |  |  |  |  |  |

1. **Please indicate Yes or No to each of the below:**

|  | Yes | No |
| --- | --- | --- |
| Do you keep samples of formula in your clinic to distribute to mothers and their babies |  |  |
| Do you have advertisement in your clinic on formula products, or well-baby care documents that have formula companies’ logo on them? |  |  |
| Do you have brochures / pamphlets in your clinic that you give to mothers about breastfeeding resources in the city |  |  |
| Do you recommend to start supplementing with formula, if a healthy term baby who is exclusively breastfed has not regained birth weight by 2 weeks |  |  |
| Do you routinely recommend to supplement with formula if a mother feels that her milk supply is inadequate |  |  |
| Do you typically recommend feeding glucose water or formula to the otherwise healthy newborn while waiting for the mother’s milk to come in |  |  |
| Do you routinely recommend that babies breastfeed on each side for 15-20 minutes every 3 hours |  |  |

**Attitudes/beliefs**

1. **Please indicate to what extent do you agree on a scale from (5) Strongly Agree to (1) Strongly Disagree on each of the below statements:**

|  | (5) | (4) | (3) | (2) | (1) |
| --- | --- | --- | --- | --- | --- |
| The child's primary physician is responsible for performing an evaluation of breastfeeding, including position, latch and milk transfer in the first 3 to 5 days after birth |  |  |  |  |  |
| As a Pediatrician, I have an influence on a mother’s decision to breastfeed her infant |  |  |  |  |  |
| It is practical for working mothers to continue to breastfeed their infants |  |  |  |  |  |
| It is acceptable for women to breastfeed in public |  |  |  |  |  |
| My residency training prepared me to support breastfeeding mothers |  |  |  |  |  |
| It’s not the pediatrician’s responsibility to follow up on breastfeeding progress and success |  |  |  |  |  |
| Mixed feeding (breastmilk and formula) is a more practical acceptable feeding method |  |  |  |  |  |
| Formula-fed babies are just as healthy as breastfed babies |  |  |  |  |  |
| A mother should stop breastfeeding once she knows she is pregnant |  |  |  |  |  |

1. **Please rate how confident / comfortable you are doing each of the below on a scale from (5) being very confident / comfortable to (1) being very unconfident/ uncomfortable**

| How confident/ comfortable are you in: | (5) | (4) | (3) | (2) | (1) |
| --- | --- | --- | --- | --- | --- |
| Teaching mothers about good breastfeeding positioning |  |  |  |  |  |
| Addressing breastfeeding related problems (engorgement, mastitis, etc..) |  |  |  |  |  |
| Evaluating whether a baby's latch is successful |  |  |  |  |  |
| Assessing whether there is good milk transfer from mother to baby during breastfeeding |  |  |  |  |  |
| Counselling mothers on cracked and sore nipples |  |  |  |  |  |
| Teaching mothers how to express milk |  |  |  |  |  |

**Knowledge:**

1. **Please answer by TRUE or FALSE**

|  | TRUE | FALSE | I don't know |
| --- | --- | --- | --- |
| Exclusive breastfeeding is recommended for the first six months of life by the World Health Organization (WHO) |  |  |  |
| It is recommended to completely wean breastfed babies from the breast at the age of 1 year, according to WHO |  |  |  |
| Solid foods introduction should start when the baby is 4 months, according to WHO |  |  |  |
| Offering the baby water during the night will help the baby hydrate and sleep better |  |  |  |
| The current infant Formulas are nutritionally equivalent to breastmilk |  |  |  |
| Formula is sometimes superior to breastmilk if it was fortified with iron and vitamin D that are both lacking in human milk |  |  |  |
| Bottles are the best option to use when breastmilk is pumped or when formula is provided |  |  |  |
| Supplementing with formula in the first weeks of life will not affect breastfeeding success |  |  |  |
| Moderate exercise in the mother can decrease quality and quantity of breastmilk |  |  |  |
| Increasing mother’s fluid or milk intake will increase her milk production |  |  |  |
| Breastfeeding has been shown to decrease the risk of SIDS |  |  |  |
| Breastfeeding is contraindicated in mothers with Hepatitis C |  |  |  |
| Breastfeeding decreases the risk of ovarian and breast cancer in mothers |  |  |  |
| Breastfeeding is safe to continue in mothers who have herpes simplex on a breast as long as the child only feeds from the unaffected breast |  |  |  |
| Breastmilk loses its core components after the baby is two years old |  |  |  |

**To your knowledge, exclusive breastfeeding consists of:**

- 1. Breast milk and occasional water or water-based fluids in hot weather.
  2. Breast milk as the main food for the infant, with other liquids or solids allowed if in very small quantities.
  3. Only breast milk, no other liquids or solids, not even water, with the exception of vitamins, minerals supplement, medicines or Oral Rehydration Solutions.
  4. I don’t know.

1. **In a baby who is breastfeeding effectively, the tongue is:**
2. Down and over the gum line
3. Positioned above the nipple
4. Against the hard palate
5. I don’t know
6. **When a breastfeeding mother complains that her nipples are sore, the first thing to do is:**
7. Assess baby’s position and latch
8. Encourage mother to continue breastfeeding as she will get used to it soon
9. Give mother a prescription for a topical anti-fungal medication
10. Recommend that mom holds off breastfeeding for a few days so that her nipples heal
11. I don’t know
12. **All of the following are signs that a baby is latched on properly except:**
13. The baby’s lips are visible and flanged outward
14. There is no clicking sound as the baby sucks
15. No part of the areola can be seen
16. Mother has no persistent nipple pain
17. I don’t know
18. **A mother complains that her 6 week old infant has been breastfeeding almost every hour for a day or two. What do you tell her?**
19. Explain that the baby requires more milk because he/she is growing and frequent breastfeeding is his/her way to increase milk supply
20. Recommend giving the infant formula to help with the frequent feedings
21. Explain that this is a sign of insufficient breast milk and should start supplementing
22. Recommend that she comes in to weigh the baby before and after a feed to see if he/she is taking enough in
23. I don’t know
24. **An otherwise healthy 5-day old breastfed infant is admitted to the hospital with jaundice. In addition to treating the child with phototherapy, you first do which of the following?**
25. Recommend offering the infant formula after every breastfeeding session
26. Recommend offering glucose water to the infant
27. Recommend more frequent breastfeeding sessions, and teach mother how and when to use a breast pump
28. Explain to the mother that she likely does not have enough milk and that she should have her partner give a few formula feeds to the baby while she gets some rest
29. I don’t know
30. **Breastfeeding initiation should be:**
31. Directly after birth
32. After the routine blood/general tests are done on the baby
33. After the mother recovers from the delivery
34. Other, please specify: _________________________

Thank you for your time and participation!
